# Preliminary comparison of the modified extraperitoneal free-PORT single incision technique and transabdominal multi-incision robot-assisted laparoscopic radical prostatectomy

**DOI:** 10.1038/s41598-023-28337-1

**Published:** 2023-01-25

**Authors:** Shangqing Ren, Yong Ou, Yaoqian Wang, Yi Wei, Cheng Luo, Bo Yang, Jiazheng Yuan, Qian Lv, Fang Zhou, Zhengjun Chen, Yu Nie, Jie Lin, Yilei Wu, Bo Yang, Shida Fan, Dong Wang

**Affiliations:** 1 Department of Robotic Minimally Invasive Surgery Center, Sichuan Academy of Medical Sciences and Sichuan Provincial People’s Hospital, Affiliated Hospital of the University of Electronic Science and Technology of China, Chengdu, 610072 China; 2 School of Medicine, University of Electronic Science and Technology of China, Chengdu, 610051 Sichuan China; 3 School of Computer Science and Engineering, University of Electronic Science and Technology of China, Chengdu, 611731 Sichuan China; 4 Department of Medical Records Statistics, Sichuan Provincial People’s Hospital, University of Electronic Science and Technology of China, Chengdu, 611731 Sichuan China; 5 Department of Paediatric Surgery, Sichuan Academy of Medical Sciences and Sichuan Provincial People’s Hospital, Chengdu, 610072 Sichuan China

**Keywords:** Oncology, Urology

## Abstract

To compare the clinical efficacy of an innovative modified single-incision technique without special extraperitoneal PORT with that of transperitoneal multi-incision robot-assisted laparoscopic radical prostatectomy and to explore the feasibility and safety of the former. A retrospective analysis was performed on 259 patients who received robot-assisted laparoscopic radical prostatectomy in the Robot Minimally Invasive Center of Sichuan Provincial People's Hospital between September 2018 and August 2021. Among them were 147 cases involving extraperitoneal single incision with no special PORT (Group A) and 112 cases involving multiple incisions by the transperitoneal method (Group B). Differences in age, PSA level, Gleason score, prostate volume, body mass index, clinical stage, lower abdominal operation history, and lymph node dissection ratio between the two groups were not statistically significant (P > 0.05). All operations were performed by the same operator. In this study, all 259 operations were completed successfully, and there was no conversion. There was no significant difference in transperitoneal blood loss, postoperative hospital stay, positive rate of incision margin, indwelling time of urinary catheter, satisfaction rate of immediate urine control, satisfaction rate of urine control 3 months after operation, positive rate of postoperative lymph node pathology or postoperative pathological stage between the two groups (P > 0.05). There were significant differences in operation time, postoperative exhaust time and incision length (P < 0.05). The modified extraperitoneal nonspecial PORT single-incision technique is safe and feasible for robot-assisted laparoscopic radical prostatectomy, and its curative effect is similar to that of transperitoneal multi-incision RARP. It has the advantages of a short operation time, less impact on the gastrointestinal tract and a more beautiful incision. The long-term effect of treatment needs to be further confirmed by prospective studies.

## Introduction

Prostate cancer is one of the most common malignant tumours in the male genitourinary system. In 2020, there were 1,414,259 cases and 375,304 deaths worldwide^1^. Epidemiological data show that the occurrence of prostate cancer has ethnic and family genetic tendencies, with the highest incidence in North America, the Caribbean and people of African descent^2^. At present, the main treatments for prostate cancer include active monitoring, radical surgery, endocrine therapy, radiotherapy and chemotherapy. Due to the high-definition magnified three-dimensional imaging system, multijoint instrument arm, and 540-degree rotation of simulated wrist instruments, the da Vinci robot system more easily shortens the urologist's learning curve and reduces the difficulty of operation than other approaches. The objectives are to achieve a more complete cure of the tumour, enable the maximum degree of sexual function preservation and maximize the retention of urine control function. Although many medical centres in the world have adopted robot-assisted laparoscopic radical prostatectomy (robotic assisted laparoscopy radical prostatectomy, RARP) as the standard procedure for the treatment of prostate cancer, they still face a series of complications in patients, such as loss of sexual function and urinary incontinence^3–5^.

The continuous update of the da Vinci robotic surgery-assisted system provides more opportunities for technological innovation in single-incision laparoscopy. Although special PORT equipment is often used to help solve space constraints, it will add an additional economic burden^6,7^. To eliminate the dependence on special PORT equipment, our centre has continually explored innovative extraperitoneal space techniques since November 2020 to implement the single-incision extraperitoneal nonspecial PORT modified RARP approach^8^. In this study, the clinical data of 259 RARP patients receiving either a single incision without special extraperitoneal PORT or multiple transperitoneal incisions in our centre between September 2018 and August 2021 were retrospectively compared to analyse the feasibility and clinical efficacy of the modified technology.

## Objects and methods

### General information

A total of 259 patients with RARP were enrolled in this study, and all operations were performed by the same surgeon. Among the patients, there were 147 cases involving no special PORT extraperitoneal single-incision RARP (Group A) and 112 cases involving multiple incisions by transperitoneal method RARP (Group B). The study was approved by the Ethics Committee of Sichuan Provincial People's Hospital, and all subjects or their relatives signed informed consent forms.

### The operation method

#### Without special PORT extraperitoneal single-incision RARP (Group A)

##### Channel establishment

**Figure 1:**
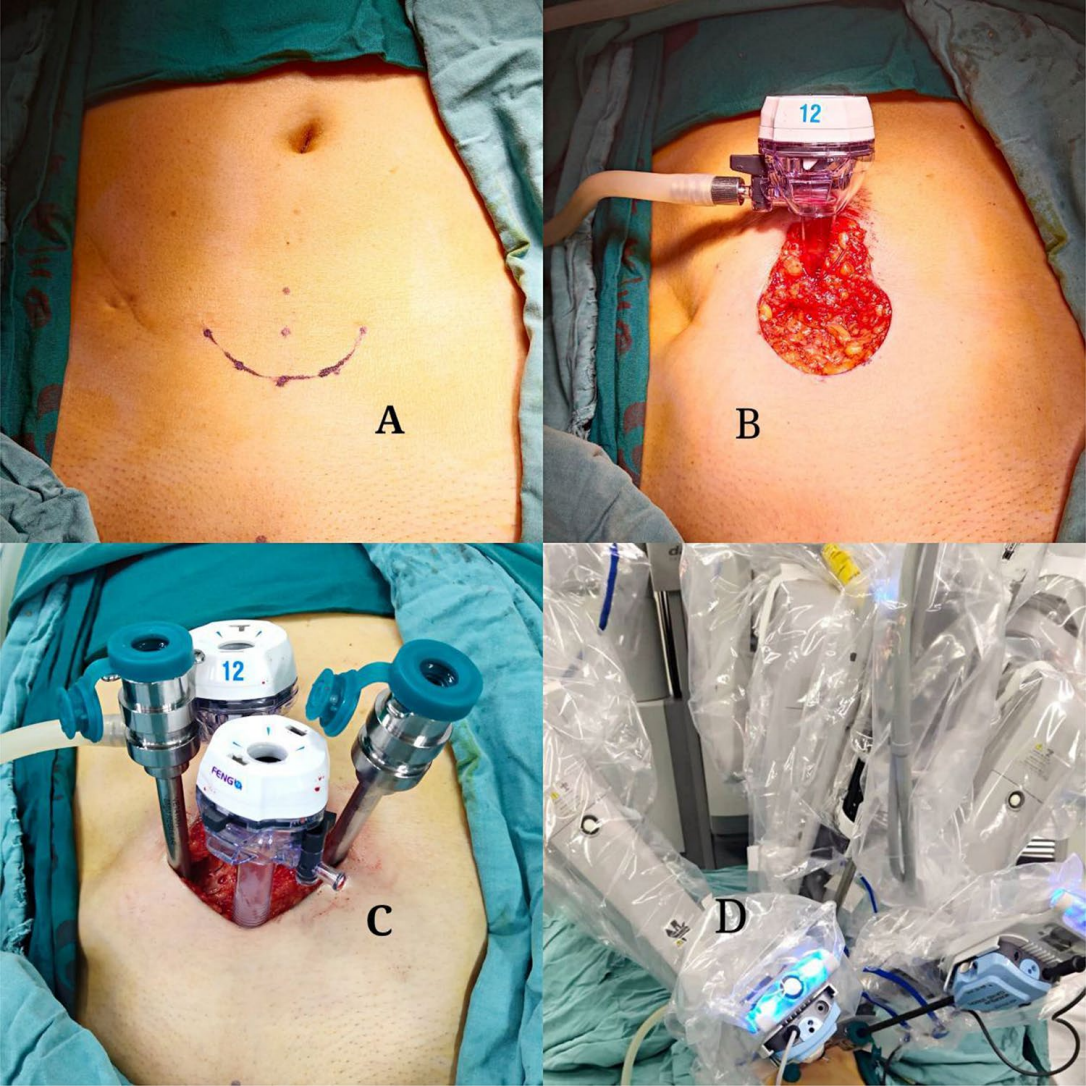
Establishment of a single-incision RARP channel with a modified extraperitoneal technique.

With the patient in the Trendelenburg position after general anaesthesia, the lowest point of the arc incision was taken from the anterior midline to 5 cm on the pubic symphysis, and 2.5 cm on both sides of the 7–8 cm midline from the pubic symphysis was used as the arc incision at both ends (Fig. 1A). The skin and subcutaneous tissue were cut in turn, the space between the subcutaneous tissue and the rectus abdominis was fully freed, and the skin flap was turned to the cephalic side and pulled. The anterior sheath of the rectus abdominis was cut longitudinally at the midpoint of the incision at 7–8 cm from the pubic symphysis, 2.5 cm was extended upwards, and the fingers were placed into the blunt separation of the rectus abdominis and the peritoneal space. The extraperitoneal space was dilated with a homemade balloon, and 900 ml gas was injected into the balloon for 10 s. The extraperitoneal space was examined, and a 12 mm trocar was placed close to the anterior sheath incision (Fig. 1B). The anterior sheath of the rectus abdominis was sutured to maintain airtightness, the space was inflated, and the lens was inserted for observation. The lower edge of the arc incision was pulled, the 12 mm trocar was placed at 3–4 cm above the pubic symphysis under direct vision, both ends of the arc incision were pulled, and two robotic metal puncture kits were placed at 3–4 cm on both sides of the midline (Fig. 1C). The da Vinci Si robot-assisted laparoscopic surgery system was connected (Fig. 1D). The distal end was the lens hole, and the proximal end was the auxiliary hole.

##### Operation procedures

**Figure 2:**
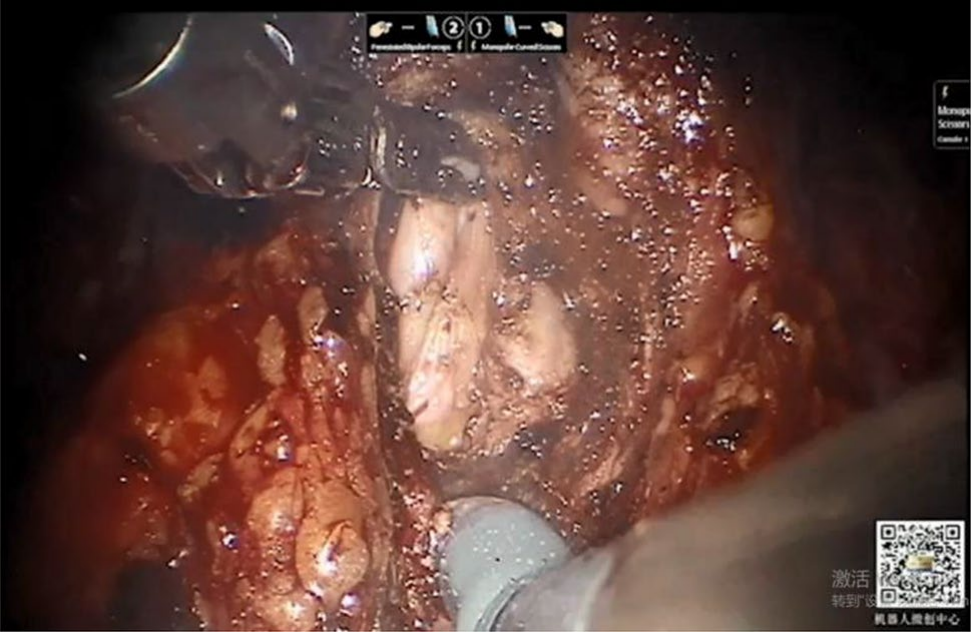
The seminal vesicles were completely exposed after the bilateral vas deferens were severed.

A 30-degree upwards visual field was used to expose the pubic symphysis area and Retzius space, free and remove the fat on the surface of the prostate, cut open the bilateral intrapelvic fascia and expose the deep dorsal vein complex of the penis (dorsal vascular complex, DVC) and pubic prostatic ligament. The junction with the prostate bladder was separated and cut by monopolar electric scissors. After confirming the posterior wall of the bladder neck and middle lobe of the prostate, the posterior wall of the bladder neck was cut open, and the muscles between the base of the prostate and detrusor of the bladder were identified for sharp separation to expose the vas deferens and seminal vesicles. The seminal vesicles were completely exposed after the bilateral vas deferens were severed (Fig. 2). The Denonvilliers’ Fascia was cut, and the dorsal side of the prostate was separated to the apex of the prostate. In all cases, the vascular nerve bundle was reserved. The lateral ligament of the prostate was ligated with HEM-O-LOK and severed. The DVC was repeatedly sutured with 2-0 barbed wire, and the sutures were temporarily left uncut and placed on the left side of the gap. The urethra was resected after dissociating the tip of the prostate, and the urethral length 1.5–2.0 cm was preserved. The bladder neck and urethra were anastomosed continuously with 2-0 double needle inverted needle thread from the posterior lip of the bladder neck. A 20F three-cavity catheter was retained. The suture of the DVC was cut and ligated after the water injection test, which showed that there was no urine leakage and no active bleeding. After quitting the instrument, the prostate specimen was removed through an arc single incision, a plasma drain was placed at the site of the vesicourethral anastomosis, and the incision was sutured layer by layer.

#### Multiple incisions by transperitoneal method RARP (Group B)

##### Channel establishment

After general anaesthesia, the disposable 12 mm cannula was placed on the upper edge of the navel, and the robot laparoscope was placed. Under direct vision, an 8 mm robotic metal cannula was placed 1.5–2.0 cm below the level of the paraumbilical region of the right left lateral rectus abdominis and 8–10 cm from the lens hole. The No. 1 and No. 2 mechanical arms were placed.

**Figure 3:**
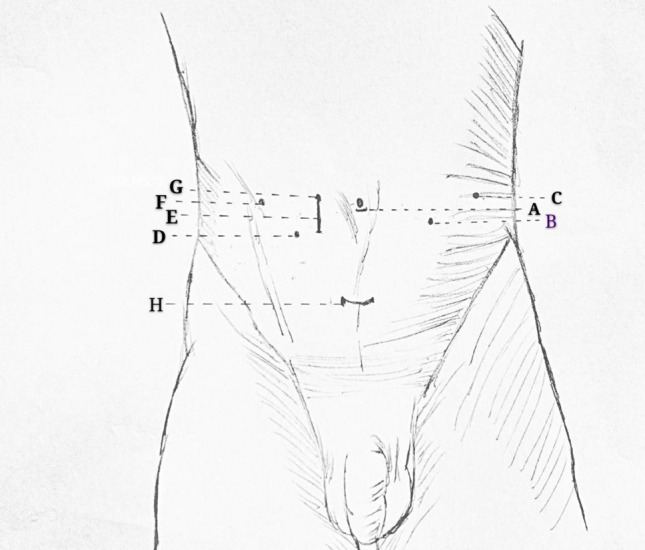
“A” shows a lens cannula, “B” shows a No. 2 arm cannula, “C” shows a No. 3 arm cannula, “D” shows a No. 1 arm cannula, “E” shows an incision for removing a specimen with a transperitoneal approach, “F and G” show auxiliary cannulas, and “H” shows an incision through an extraperitoneal single-hole pathway.

The 8 mm casing was placed 1.5–2.0 cm above the No. 2 manipulator, and the No. 3 manipulator was placed on the left axillary front line of the No. 2 arm 8–10 cm. The 12 mm cannula was placed as the helper hole 4 cm on the right side of the umbilical plane lens hole and 4 cm on the outside of the right mechanical arm, and the operation was performed using a transperitoneal approach (Fig. 3).

##### Operation procedures

**Figure 4:**
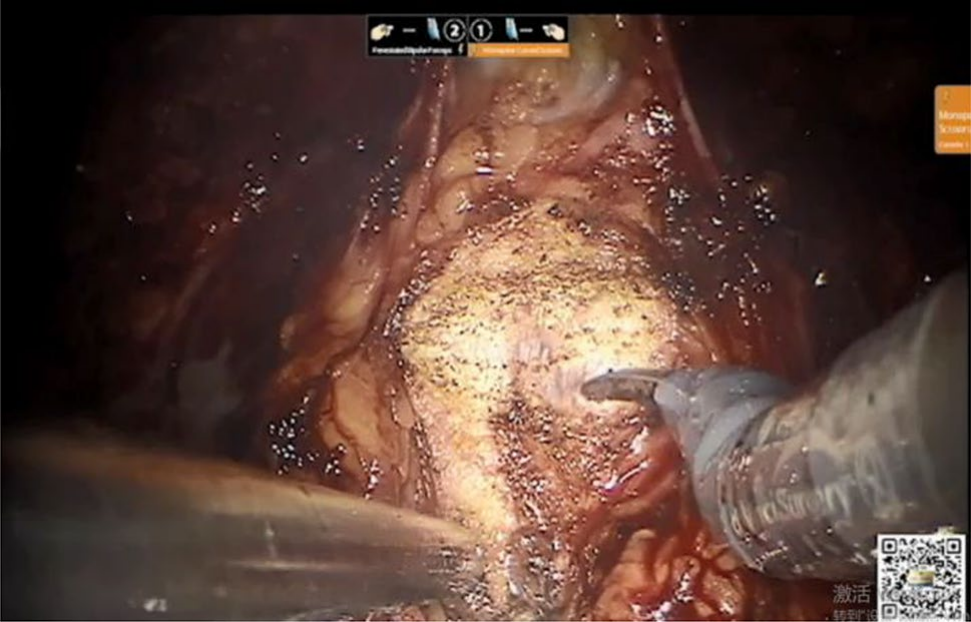
Amputation of the bladder neck.

First, the seminal vesicle gland and vas deferens were dissociated, the Dirichlet space was established, and the space was filled with gauze as a mark. With the inverted U-shaped peritoneal incision along the median umbilical ligament, the anterior bladder space was entered, the fascia around the prostate and as far as the tip was freed, the intrapelvic fascia was opened, both sides of the prostate were dissociated until the deep dorsal vein complex (dorsal vein complex, DVC) was clearly exposed, and the pubic prostatic ligament was retained. After amputation of the bladder neck (Fig. 4), the lateral prostatic ligament was dissected, and the bilateral ligament was ligated. All of them were tied to preserve the neurovascular bundle at the tip of the prostate. The prostate was completely removed after cutting the DVC with 2-0 absorbable sutures. After the bladder neck was anastomosed to the urethra, a 20F three-lumen catheter was retained. After ensuring that there was no water leakage at the anastomosis, the specimen was removed from the instrument, a plasma drain was placed at the site of the vesicourethral anastomosis, and the incision was sutured layer by layer.

### Observation index

The operation time, intraoperative blood loss, postoperative hospital stay, postoperative exhaust time, positive rate of incisal margin, indwelling time of urinary catheter, erectile function, immediate urine control satisfaction rate (24 h using urine pad was ≤ 1 as urine control satisfaction rate), postoperative 3-month urine control satisfaction rate, postoperative lymph node pathology, incision length and biochemical recurrence rate were compared between the two groups.

### Statistical methods

SPSS 21.0 software was used to input and analyse the data. Age, operation time, postoperative hospital stay, postoperative exhaust time, catheter indwelling time and incision length were observed to be in accordance with a normal distribution, expressed as “x ± s,” and compared between the two groups by independent sample t test. PSA, prostate volume and intraoperative bleeding volume had skewed distributions, expressed by M (Q1 and Q3), and the Mann‒Whitney U test was used to compare the two groups. The adoption rate/constituent ratio, frequency of classified data such as Gleason score, BMI, clinical stage, pathological stage, history of lower abdominal operation, lymph node dissection, positive cutting edge, preservation of erectile function after operation, satisfactory urine control immediately after operation, satisfactory urine control 3 months after operation, positive lymph node pathology and biochemical recurrence 3 months after operation (PSA was higher than 0.2 ng/mL), and chi-square test were used to compare between groups (two-sided test, test level α = 0.05).

### Declaration

This study complies with the principles of the Helsinki Declaration and the relevant ethical requirements of Sichuan Academy of Medical Sciences and Sichuan Provincial People's Hospital (ethics No. 100, 2020).

## Results

In this study, all 259 operations were completed successfully, and there was no conversion. There was no significant difference in age, PSA level, Gleason score, prostate volume, body mass index (BMI), clinical stage, history of lower abdominal operation or proportion of lymph node dissection between the group A and group B. General information is shown in Table 1. The observation indexes of group A and group B were as follows, respectively: the intraoperative bleeding volume was 119.35 (50.0–180.0) ml and 116.43 (50.0–200.0) ml; the postoperative hospital stay was 10.18 (6.0–15.0) days and 9.8 (5.0–23.0) days; the number of patients with positive incision margins was 20 (13.6%) and 18 (16.1%); the postoperative indwelling times of the urinary catheter were 8.23 (5.0–14.0) days and 8.25 (5.0–15.0) days; the postoperative erectile function was preserved in 16 cases (10.9%) and 15 cases (13.4%); the number of satisfactory cases of immediate urinary control after operation was 88 (60.5%) and 60 (53.6%); the number of satisfactory cases of urinary control 3 months after the operation was 133 (90.5%) and 102 (91.1%); the number of pathologically positive cases of postoperative lymph nodes was 1 case (0.7%) and 0 cases (0%). Three months after the operation, the number of biochemical recurrence or persistent PSA status was 5 (3.4%) and 3 (2.7%). The numbers according to postoperative pTNM stage were as follows, respectively: pT2: 137 (93.2%), 105 (93.7%) and pT3a: 10 (6.8%), 7 (6.3%). There was no significant difference in the above indexes (P > 0.05). The operation times, respectively, were 132.63 (80.0–200.0) min and 143.82 (100.0–202.0) min; the postoperative exhaust times were 2.88 (2.0–4.0) d and 1.3 (1.0–4.0) days. The incision length was 5.32 (4.1–6.5) cm and 8.07 (6.5–9.0) cm, respectively, and the above indicators were statistically significant (< 0.05). Single-incision RARP without special extraperitoneal PORT was superior to traditional transperitoneal multi-incision RARP in operation time, postoperative exhaust time and incision aesthetics. The results are shown in Table 2.

**Table 1:** General information.

	Group A (n = 147)	Group B (n = 112)	P
Age, years, mean (SD)	71.05 (7.82)	70.89 (7.30)	0.871
BMI, kg/m^2^, mean (SD)	23.60 (3.69)	23.38 (3.07)	0.598
Preoperative serum total PSA, ng/mL, mean (SD)	22.21 (20.22)	24.67 (20.17)	0.332
Prostate volume, mL, mean (SD)	47.16 (25.92)	49.00 (22.91)	0.554
Gleason score			0.647
6	20 (13.6%)	21 (18.8%)	
7	77 (52.4%)	46 (41.1%)	
8	16 (10.9%)	31 (27.7%)	
9	34 (23.1)	14 (12.5%)	
cTNM stage, n (%)			0.352
cT1c	1 (0.7%)	3 (2.7%)	
cT2a	27 (18.4%)	30 (26.8%)	
cT2b	50 (34.0%)	27 (24.1%)	
cT2c	62 (42.2%)	47 (42.0%)	
cT3a	7 (4.8%)	5 (4.5%)	
History of lower abdominal operation	7 (4.8%)	6 (5.4%)	0.976
Proportion of lymph node dissection, n (%)	7 (4.8%)	5 (4.5%)	0.910

*BMI* body mass index, *SD* standard deviation.

**Table 2:** Comparison of postoperative efficacy of patients (cases).

	Group A (n = 147)	Group B (n = 112)	P
Operative time, min, mean (SD)	132.63 (32.17)	143.82 (26.47)	0.003
Estimated blood loss, mL, mean (SD)	119.35 (23.62)	116.43 (33.92)	0.437
Open conversion, n (%)	0 (0%)	0 (0%)	
Postoperative hospital stay, days	9.80 (2.89)	10.18 (2.15)	0.232
Postoperative exhaust time, days	1.87 (0.772)	2.88 (0.64)	0.000
Positive lymph nodes, n (%)	1 (0.7%)	0 (0%)	0.252
Positive surgical margin, n (%)	20 (13.6%)	18 (16.1%)	0.579
Postoperative indwelling time of urinary catheter, days	8.23 (1.84)	8.25 (1.70)	0.933
pTNM stage, n (%)			0.125
pT2	137 (93.2%)	105 (93.7%)	
pT3a	10 (6.8%)	7 (6.3%)	
Postoperative erectile function, n (%)	16 (10.9%)	15 (13.4%)	0.539
Immediate urinary control after operation, n (%)	88 (60.5%)	60 (53.6%)	0.312
Urinary control 3 months after operation, n (%)	133 (90.5%)	102 (91.1%)	0.870
Biochemical recurrence or persistent PSA status after 3 months, n (%)	5 (3.4%)	3 (2.7%)	0.740
Incision length, cm	5.32 (0.56)	8.07 (0.48)	0.000

A PSA above 0.2 ng/ml at 3 months postoperatively was defined as biochemical recurrence or persistent PSA status. Erectile function was defined as a patient's postoperative IIEF scale score greater than 12 and most of the time being able to complete sexual life.

*SD* standard deviation.

## Discussion

Since urology single-hole laparoscopy was introduced into China in 2008, it has experienced different periods of development in the urinary field, while the development of a special multichannel device, namely, commercial PORT, has promoted the development of single-hole technology. However, due to the disappearance of the operating triangle of the main instrument, the impact of endoscopy with other instruments during the operation still limits the popularization of this technique to a great extent. At present, the development of single-hole technology in China is becoming increasingly stable, and the surgical robot system has brought a new development direction for urologists. Professor Ren gave full play to the high flexibility and fine operation ability of the robot system in 2018 and completed the first single-hole RARP in Asia^9^.

To solve the problem of instrument collision, foreign experts and scholars actively explores new solutions. From a homemade single-hole casing for early gloves to a commercial single-hole special casing R-port, SILS Port, QuadPort, TriPort, LagiPort and so on, special endoscopic operation platforms and instruments were developed, which solved the problem of mutual interference of single-hole instruments to a certain extent. The progress of minimally invasive technology has also prompted domestic and foreign experts and scholars to constantly explore the comparison of the clinical effects of RARP under various new technologies. Since November 2020, to eliminate the dependence on PORT and reduce the cost of treatment, our centre began to try a modified extraperitoneal technique, that is, a single incision extraperitoneal technique without a special PORT. In this study, there was no significant difference in intraoperative blood loss, postoperative hospital stay, postoperative exhaust time, positive rate of incisal margin, indwelling time of urinary catheter, postoperative erectile function, immediate postoperative urine control satisfaction rate, postoperative 3-month urine control satisfaction rate or postoperative 3-month biochemical recurrence or persistent PSA status rate between the modified extraperitoneal technique and traditional transperitoneal RARP, which was consistent with the results of previous foreign studies^10,11^.

The latest research results show that there is no significant difference in perioperative and pathological results between single-incision RARP and standard laparoscopic multiple-incision RARP. Some studies have reported that single-incision surgery may have more advantages in the recovery of postoperative erectile function^12–14^, but there are also reports that single-incision surgery takes a longer time because of the establishment of space^15,16^. This is because although the robot system multijoint instrument arm and simulated wrist instrument improve the problem of too narrow operation space to some extent, the collision between robotic arms will still occur during placement of a slightly large free suture, which also makes it more difficult for assistants to cooperate. Previous studies suggest that compared with traditional transperitoneal multi-incision RARP, single-incision extraperitoneal RARP has a shorter hospitalization time, less demand for postoperative painkillers and anaesthetics, and a considerable incidence of postoperative complications and readmission rate^17^. Some studies suggest that the use of the da Vinci SP platform operation can classify RARP as daytime surgery and can reduce or avoid the use of analgesics. Short-term function and oncology results are not significantly different compared to routine hospitalization^18^. Some scholars believe that the single-incision scar has the highest score in terms of psychosocial impact and aesthetics, which will have a great impact on the quality of life of patients^19,20^.

Transperitoneal RARP can delay the recovery of digestive tract function due to interference with the gastrointestinal tract and even has the possibility of postoperative intestinal obstruction and abdominal adhesion, so previous abdominal surgery is a relative contraindication^21^, while the extraperitoneal approach can expand the scope of surgical indications on the premise of a good cosmetic effect and reduce incision pain. However, due to the significant regional differences in the incidence of prostate cancer in China and the imbalance of diagnosis and treatment levels and economic conditions in different regions, minimizing the medical expenses of patients is also one of the goals pursued by surgeons on the premise of minimal invasiveness. Therefore, the modified technology in this study was intended to eliminate the restrictions of PORT. The results show that this technology is a new surgical method worthy of implementation; this approach also makes up for the disadvantage that the development of single-incision technology is restricted by the lack of special PORT equipment in some medical institutions.

At present, the modified methods of RARP are also diversified, and the latest research is mainly focused on different approaches to retain Retzius^22–26^. The results suggest that RS-RARP (Retzius sparing) had better postoperative continence recovery than C-RARP (traditional), while sexual function recovery rates were not significantly different. There were also no significant differences in operation time, intraoperative blood loss, length of stay, positive margin rate or complications^22^. Additionally, a report showed that compared with C-RARP, RS-RARP showed better recovery of continence, shorter console time, and a lower incidence of hernia. Although there was no significant difference in overall PSM, we suggest that the surgeon should be more careful if the lesion is in the anterior prostate^23^. However, some studies are also controversial. RS-RARP improves early urinary continence recovery compared to anterior RARP, with this advantage being lost after 3 to 6 months. However, erectile function and quality of life were comparable between the two techniques. The results concerning the rate of positive margins remain controversial. Future studies with longer follow-up are needed to better assess oncologic outcomes^24^. The application of a transvesical approach to RARP for localized PCa could obtain promising outcomes in terms of postoperative UC recovery. In addition, surgical strategies encompassing the nerve-sparing technique and the Retzius-sparing procedures during RARP, namely, the transvesical or posterior approach, could independently enable early achievement of postoperative continence^25^.

At the same time, this study has some limitations. First, subjective factors interfere with the selection of patients in the retrospective study. Second, the modified technique group was completed on the experience of the transperitoneal group, and the surgical experience was more abundant for the chief surgeon when operating on patients in the extraperitoneal group than when operating on patients in the transperitoneal group. This may be one of the reasons for the difference in operation time. Finally, the extraperitoneal single-hole technique limits the scope of standard pelvic lymph node dissection. In this study, obturator lymph node dissection was performed in the extraperitoneal single-hole group, and standard pelvic lymph node dissection was performed in the transperitoneal group. The results showed that 1 case (0.7%) was positive in the extraperitoneal single-hole group, and there was no significant difference between the extraperitoneal group and the transperitoneal group. Therefore, in the future, prospective control studies are still needed for verification, and we also need to find better methods to expand the surgical space and scope of operation.

It is worth noting that there is a possibility of peritoneal rupture leading to failure of space establishment at the initial stage of learning from the extraperitoneal single incision channel, but the problem will be resolved as experience accumulates.

Single-incision robot-assisted laparoscopic radical prostatectomy without a special PORT is safe and feasible, and its curative effect is similar to that of the traditional multiple-incisions approach, with the advantages of a short operation time, less influence on the gastrointestinal tract and a more beautiful incision. The long-term effect of treatment needs to be further confirmed by prospective studies.

## Date availability

The raw data supporting the conclusions of this article will be made available by the authors without undue reservation. Please contact the author Yong Ou (email: 675893648@qq.com).
